# Supplementary material for: Susceptibility of sheep to experimental co-infection with the ancestral lineage of SARS-CoV-2 and its alpha variant
Source: Emerg Microbes Infect. 2022 Feb 24;11(1):662–75. doi: 10.1080/22221751.2022.2037397 (PMC8881078; doi:10.1080/22221751.2022.2037397)
Supplement: Supplemental Material [file TEMI_A_2037397_SM5108.docx]

**SUPPLEMENTARY FIGURES 1**


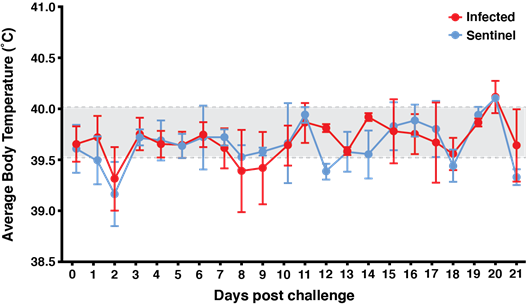


**Supplementary Figure 1. Body temperature.** Daily average rectal temperatures of sheep inoculated with SARS-CoV-2 (red) and co-mingled sentinel sheep (blue) showed no significant change over the course of the experiment. The baseline temperature (grey area; 39.5°C to 40°C) was determined from all sheep before infection.

**SUPPLEMENTARY TABLES 1-2**

| **Supplementary Table 1. Virus isolation from swabs and tissues.** | | | |
| --- | --- | --- | --- |
| *DPC* | *Sample Type* | *Animal Number* | *Virus titer (TCID_50_/mL)* |
| 1 | Nasal swab | 713, 714, 715, 717, 719 | NEG |
|  | Oral swab | 719 | NEG |
| 3 | Nasal swab | 715 | NEG |
| 4 | Nasal wash | 712, 713, 714 | NEG |
|  | BALF | 714 | NEG |
|  | Conchae | 712 | NEG |
|  | Ethmoturbinates | 712, 713 | NEG |
|  | Nasopharynx | 712, 714 | NEG |
|  | Trachea | 712, 713, 714 | NEG, 5.00E+00*, NEG |
|  | Bronchi | 712, 713, 714 | NEG |
|  | Lung | 714 | NEG |
|  | Tonsil | 712, 713, 714 | NEG |
|  | Retropharyngeal lymph node | 712, 713 | NEG |
|  | Tracheobronchial lymph node | 712, 713, 714 | NEG |
|  | Mesenteric lymph node | 712, 713 | NEG |
| 8 | Nasopharynx | 715, 716, 717 | NEG |
|  | Trachea | 715, 716, 717 | NEG |
|  | Tonsil | 716, 717 | NEG |
|  | Retropharyngeal lymph node | 715, 716, 717 | NEG |
|  | Tracheobronchial lymph node | 715 | NEG |
| 21 | Nasopharynx | 719 | NEG |
|  | Retropharyngeal lymph node | 718, 719 | NEG |
|  | Tracheobronchial lymph node | 719 | NEG |
| **virus isolated from rostral and proximal trachea samples of sheep #713* | | | |

| **Supplementary Table 2. Viral RNA detected in biological fluids collected from SARS-CoV-2 infected sheep.** | | | |
| --- | --- | --- | --- |
| *Necropsy* | *Sheep ID* | *Biological* | *Mean CN/ML* |
| 4 DPC | 712 | BALF | ND |
|  |  | Nasal wash | 8.21E+04 |
|  |  | CSF | ND |
|  | 713 | BALF | 3.32E+03 |
|  |  | Nasal wash | 1.35E+05 |
|  |  | CSF | ND |
|  | 714 | BALF | 5.45E+04 |
|  |  | Nasal wash | 3.37E+04 |
|  |  | CSF | ND |
| 8 DPC | 715 | BALF | ND |
|  |  | Nasal wash | ND |
|  |  | CSF | ND |
|  | 716 | BALF | 3.08E+03* |
|  |  | Nasal wash | ND |
|  |  | CSF | ND |
|  | 717 | BALF | ND |
|  |  | Nasal wash | ND |
|  |  | CSF | ND |
| 21 DPC | 710 | BALF | ND |
|  |  | Nasal wash | ND |
|  |  | CSF | ND |
|  | 711 | BALF | ND |
|  |  | Nasal wash | ND |
|  |  | CSF | ND |
|  | 718 | BALF | ND |
|  |  | Nasal wash | ND |
|  |  | CSF | ND |
|  | 719 | BALF | ND |
|  |  | Nasal wash | ND |
|  |  | CSF | ND |
| **1 out of 2 RT-qPCR reactions above the limit of detection; DPC=days post challenge; CN=RNA copy number; ND=not detected; BALF=bronchoalveolar lavage fluid; CSF= cerebral spinal fluid* | | | |
